# Asthma and Attendance in Urban Schools

**DOI:** 10.5888/pcd16.190074

**Published:** 2019-10-31

**Authors:** Sara B. Johnson, Paul Spin, Faith Connolly, Marc Stein, Tina L. Cheng, Katherine Connor

**Affiliations:** 1 Johns Hopkins School of Medicine, Department of Pediatrics, Baltimore, Maryland; 2 Johns Hopkins Bloomberg School of Public Health, Department of Population, Family and Reproductive Health, Baltimore, Maryland; 3 Johns Hopkins Bloomberg School of Public Health, Department of Mental Health, Baltimore, Maryland; 4 Johns Hopkins School of Education, Baltimore, Maryland; 5 Baltimore Education Research Consortium, Baltimore, Maryland

## Abstract

**Introduction:**

Asthma is linked to student absenteeism, a risk factor for poor achievement and school dropout. Studies of asthma and absenteeism have common limitations, including relying on parent-reported asthma, which may be unreliable and prone to selection, and inadequately accounting for confounding health and social risks. Therefore, the rate of absenteeism attributable to asthma and the extent to which better asthma control would translate into better attendance remain unclear.

**Methods:**

Participants were 1,194 students in 2 large urban US schools (1 elementary, 1 middle) in 2016–2018. Student asthma was assessed based on parent report on health forms, student-reported asthma-related emergency department/hospitalization or medication use, and school health center record of asthma. Multiple imputation was used to reduce selection from missing asthma reports. The relationship between asthma and school district–reported days absent was estimated using Poisson random intercept regression, accounting for health and demographic covariates.

**Results:**

Parent-reported ever asthma (27%) was not associated with absenteeism in adjusted models. Student-reported asthma health care or medication use (16%) and school health center record of asthma (17%) were associated with higher absenteeism (incidence rate ratio [IRR], 1.16; 95% confidence interval [CI], 1.01–1.35 and IRR, 1.21; 95% CI, 1.09–1.34, respectively). Student-reported asthma and school health center record of asthma were associated with 1.9 and 1.5 absences per year, respectively.

**Conclusion:**

Student-reported and school health center record of asthma explained 14% to 18% of student absenteeism, even after accounting for other health and social risks. When possible, student reports should supplement parent reports to ensure that students with asthma are identified and obtain access to care.

**Summary**

**What is already known on this topic?**

Asthma is linked to student absenteeism. Previous studies are limited by parent report of asthma and inadequately accounting for health and social risks that affect asthma and absenteeism.

**What is added by this report?**

Asthma reported by students and the school-based health center, but not by parents, was associated with more absenteeism, even after accounting for health and social factors. Asthma explained 14% to 18% of absenteeism.

**What are the implications for public health practice?**

Supplementing parent-reported asthma with student reports could improve case finding and improve estimates of the burden of asthma on student attendance.

## Introduction

Approximately 1 in 101 schoolchildren in the United States has asthma (1). Forty-nine percent have missed 1 or more days of school due to the condition (2). Absenteeism, in turn, is associated with lower grades and assessment scores (3).

Asthma is more prevalent among low-income and racial/ethnic minority children and children in urban areas (4–6). Disparities in asthma control are related to access to and quality of health care, adherence to medication use, and social factors such as segregation and psychosocial stressors (7). These factors also contribute to absenteeism (8). However, many studies of asthma and attendance have not accounted for factors such as poverty, access to transportation, and comorbid health conditions (8,9). It is unclear, therefore, whether improved asthma management alone would meaningfully improve attendance among low-income and racial/ethnic minority students with asthma (9).

Many studies of asthma and attendance have relied on parent reports (9), which may be unreliable in settings with high asthma prevalence (10,11), and may underestimate asthma among low-income children because of difficulty reaching parents (11,12). Thus, to characterize the public health burden of asthma in urban schools for planning and intervention, supplements to parent reports are needed.

We examined asthma and attendance in 2 large urban schools. First, we investigated schoolwide prevalence of 3 asthma indicators based on 1) parent reports; 2) student reports of asthma-related emergency department (ED) visits, hospitalization, or use of asthma medication; and 3) school health center record of asthma. We expected prevalence would be highest based on parent reports, which were expected to be most inclusive. Second, we investigated the relationship between each asthma indicator and attendance, accounting for health and sociodemographic factors. We hypothesized that the relationship would be strongest for student reports and that all relationships would be attenuated after accounting for health and social factors.

## Methods

### Participants and setting

Participants were 1,194 students who attended 2 large urban schools (1 elementary, 1 middle) for the 2016–2017 (school year 1 [SY1]) and 2017–2018 (SY2) school years.

The Title I public elementary (grades K–4) and middle school (grades 5–8) share 1 building. A health center is located onsite and provides school nursing and clinician-directed comprehensive chronic disease management through a school-based health center (SBHC). Of all students, 99% are African American and approximately 80% qualify for free and reduced-price meals. Students are enrolled in the schools by lottery and come from neighborhoods across the school district.

### Measures

**Absenteeism.** The total number of absences during each school year was calculated using school district records. On each school day, the schools recorded whether, and for how long, a student was absent. All absences regardless of duration were aggregated (>99% of absences were recorded as full-day absences).

**Risk of asthma.** Three data sources were used to characterize asthma.

1) Parent-report of ever asthma (n = 926). Parents indicated whether their child had ever had an asthma diagnosis (yes/no) on the schools’ student health form. Seventy-seven percent of parents returned a school health form in at least 1 of the 2 years.

2) Student-report of asthma-related ED visit, hospitalization, or asthma medication use (n = 731). Because of incomplete school health form data, school nurses conducted schoolwide asthma screening to determine number of cases. All students were asked to complete 2 questions adapted from Redline et al (13). They answered yes or no based on the last 12 months to 1) “I take medication for asthma” and 2) “I went to the hospital or emergency room because I had trouble breathing.” The questions were administered one time by school nurses; missing data were due primarily to homeroom teacher or student absence or tardiness or to incomplete responses. The student-reported asthma-related ED visit/hospitalization and asthma medication use questions were validated using school health center records of asthma as the gold standard.

3) School health center record of asthma (n = 1,194). Students were classified as having asthma if they had asthma noted in their school nursing record (regardless of severity or persistence), had a rescue or controller medication order on file with the school nurse, or had sought care for asthma in the SBHC in either year of the study.

**Covariates.** Factors that could confound the relationship between asthma and attendance were included in statistical models: grade, sex (male/female), tenure in school, chronic health condition other than asthma (based on health center records, eg, diabetes, seizure disorder), allergies (yes/no), mental/behavioral health problem (yes/no), overweight/obese (yes/no, based on age- and sex-adjusted body mass index [BMI], measured by trained staff and calculated using 2000 Centers for Disease Control and Prevention growth charts [14], and enrollment in the SBHC (yes/no). In addition, area-based measures were calculated at the census-tract level using home addresses to account for transportation challenges: distance from home to school in miles, proportion of households without a private vehicle, and the interaction between distance from school and private vehicle ownership. Census-tract poverty rate was used as a proxy for student socioeconomic status (SES) because individual indicators were not systematically collected by the school. Studies suggest that individual and area-based measures of SES are moderately to highly correlated (15,16).

### Statistical analysis

Missing data ranged from 11% (neighborhood-level covariates) to 39% (student reports of asthma-related ED use or asthma medication use). After confirming that patterns of missing data were consistent with the missing-at-random assumption (ie, missingness due to a predicable reason [17]; in this case, missingness was uncorrelated with asthma prevalence after accounting for the influence of observed variables), missing data were multiply imputed using chained equations (18,19). One hundred imputed data sets were generated based on a 2-stage quadratic rule to achieve a prespecified level of replicability of coefficient standard errors. The imputation model included all covariates as well as monthly counts of absences (19,20), which were associated with health form nonreturn, student asthma question completion, and BMI. Number of days enrolled at the school from September to November during SY1, which was highly correlated with missingness on BMI and the student-reported asthma-related ED/hospitalization or asthma medication use measures, was included as an auxiliary variable. Results from models using nonimputed data and imputed data were qualitatively similar.

Poisson random intercept regression models were used to examine the relationship between each asthma indicator and the log of annual absences, controlling for observed confounders. Because approximately 6% of students enrolled after the start of SY1, the log of days enrolled was included as a linear offset. In preliminary analyses, differences in the relationship between asthma and attendance across years were tested using a school × asthma interaction term; because the relationship was similar across years, the 2 years were pooled. Model coefficients were transformed into incidence rate ratios (IRRs) that compared absenteeism of students with asthma with absenteeism of students without asthma. Analyses were conducted using Stata/SE version 15.1 (StataCorp LLC). The study was approved by the university and school district institutional review boards.

## Results

### Validation of student reports

Together, student-reported ED visits/hospitalization for asthma and student-reported asthma medication had low sensitivity (43% true positive rate) and high specificity (93% true negative rate) (Appendix). These questions correctly predicted asthma 78% of the time and the absence of asthma 93% of the time.

### Asthma prevalence by indicator

Parent-, student-, and health center–derived asthma indicators were moderately correlated (0.41–0.66) and exhibited moderate internal consistency (Cronbach α = 0.73) (Table 1). Parent-reported ever asthma prevalence was 27%, student-reported asthma-related ED visit/hospitalization or asthma medication use prevalence was 16%, and school health center record of asthma prevalence was 17%. The asthma indicators were comparable with respect to sex, weight status, and neighborhood poverty, but students with asthma had higher rates of chronic conditions and allergies compared with students without asthma (Table 2).

**Table 1:** Correlations Between Data Sources About Reporting of Student Asthma, 2 Large Urban US Schools (N = 1,194), 2016–2018^a^

Variable	Parent Report, Mean *r* (Minimum–Maximum)	Student Report, Mean *r* (Minimum–Maximum)	Health Center Record
Parent-reported ever asthma	1 [Reference]	—	—
Student-reported asthma-related ED visit/hospitalization or asthma medication use	0.41 (0.32–0.48)	1 [Reference]	—
School health center record of asthma	0.66 (0.63–0.69)	0.44 (0.33–0.52)	1 [Reference]

Abbreviations: —, not applicable; ED, emergency department.

a Estimates are the mean (minimum–maximum) of Pearson correlations across all 100 multiply-imputed data sets. Cronbach α for parent report, child report, and health center record of asthma was 0.73.

**Table 2:** Demographic and Health Correlates of Student Asthma Indicators, 2 Large Urban US Schools (N = 1,194), 2016–2018^a^

Indicator	No Asthma (n = 789, 66%)	Parent-Reported Ever Asthma (n = 324, 27%)	Student-Reported Asthma Health Care or Medication Use (n = 187, 16%)	School Health Center Record of Asthma (n = 207, 17%)
	
No. of absences per year (standard deviation)^b^	9.84 (8.72)	11.43 (8.98)	11.69 (10.09)	12.32 (8.85)
Female	0.53 (0.52)	0.43 (0.52)	0.53 (0.60)	0.43 (0.50)
Middle school^c^	0.42 (0.69)	0.38 (0.52)	0.43 (1.22)	0.35 (0.48)
2nd year + tenure^d^	0.81 (0.99)	0.76 (0.46)	0.75 (1.98)	0.80 (0.40)
Chronic health condition other than asthma	0.25 (0.52)	0.38 (0.54)	0.33 (0.77)	0.36 (0.52)
Mental/behavioral health condition	0.12 (0.43)	0.17 (0.43)	0.16 (0.57)	0.16 (0.41)
Allergies	0.34 (0.57)	0.64 (0.54)	0.56 (0.89)	0.65 (0.53)
Enrolled in school-based health center	0.76 (0.50)	0.82 (0.45)	0.86 (0.68)	1.00 (0.07)
Overweight/obese	0.45 (0.59)	0.49 (0.59)	0.46 (0.64)	0.45 (0.54)
Neighborhood poverty^e^	0.25 (0.13)	0.26 (0.13)	0.25 (0.13)	0.26 (0.11)
Households without a private vehicle^f^	0.35 (0.16)	0.36 (0.16)	0.37 (0.17)	0.36 (0.15)
Distance to school (log miles)	0.57 (1.02)	0.57 (1.05)	0.62 (1.08)	0.61 (0.88)

a Values are percentages (standard deviations) unless otherwise indicated.

b Mean annual absences, years 1 and 2.

c Grade level was used in analytic models; however, for parsimony, the proportion of students in middle school (grades 5–8) is shown.

d 2nd year + tenure reflects the proportion of students who attended the school for at least 2 years to account for exposure to the school-based health program.

e Reported mean of family poverty is within the top 30% of census tract poverty rates (ie, the poorest 30%).

f Estimated at census-tract level associated with student’s address.

### Absenteeism by asthma indicator

Overall, students in the sample were absent 10.4 days per year. Students with asthma missed more days than students without asthma. Excess absenteeism was highest based on student-reported asthma-related ED visit/hospitalization or asthma medication use (1.9 more days that those without student-reported asthma treatment). Excess absenteeism was 1.5 days for school health center record of asthma and 1.6 days for parent-reported ever asthma.

Table 3 summarizes IRRs comparing school absenteeism for parent-reported ever asthma, student-reported asthma-related ED visit/hospitalization or asthma medication use, and school health center record of asthma. Parent reports were associated with higher absenteeism in the unadjusted model, but this relationship was not significant after adjustment. In contrast, after adjusting for covariates, student reports (IRR, 1.16; 95% CI, 1.01–1.35) and school health center record of asthma (IRR, 1.21; 95% CI, 1.09–1.34) remained associated with absenteeism. School health center record of asthma was associated with more absenteeism compared with parent reports of ever asthma (IRR, 1.09; 95% CI, 0.98–1.22), but other pairwise comparisons were similar. The magnitude of the IRRs was not attenuated by adjusting for covariates. No significant differences were found in the relationship between asthma and attendance across middle and elementary schools.

**Table 3:** School Absenteeism Among Students With and Without Asthma, by Reporter, 2 Large Urban US Schools (N = 1,194), 2016–2018^a^

Reporter	Unadjusted		Adjusted	
	IRR (95% CI)	*P* Value	IRR^b^ (95% CI)	*P* Value
Parent-reported ever asthma (27%)	1.13 (1.02–1.26)	.02	1.09 (0.98–1.22)	.12
Student-reported asthma-related ED visit/hospitalization or asthma medication (16%)	1.15 (0.99–1.32)	.06	1.16 (1.01–1.35)	.04
School health center record of asthma (17%)	1.21 (1.09–1.34)	<.001	1.21 (1.09–1.34)	<.001

Abbreviations: CI, confidence interval; ED, emergency department; IRR, incidence rate ratio.

a Poisson models were specified using an exposure variable equal to the log of school days that a student was registered during a school year.

b Adjusted for grade, sex, comorbid chronic condition, allergies, mental/behavioral health condition, overweight/obese status, enrollment in the school-based health center, distance from home to school, interacted with percentage of households in neighborhood with a private vehicle, and neighborhood poverty rate.

## Discussion

This study sought to advance our understanding of the relationship between student asthma and school absenteeism in a setting where students are at high demographic risk for both outcomes. Many studies have been limited by parent survey–based ascertainment of student asthma, which may introduce selection biases when forms are missing. To address this limitation, we evaluated student reports of asthma-related ED visit/hospitalization or asthma medication use and school health center record of asthma as adjuncts to parents’ reports of their child ever being diagnosed with asthma, and we used multiple imputation, a modern missing data method, to address potential selection due to nonresponse. Moreover, we accounted for student comorbid health conditions and social risk factors that could confound this relationship and potentially overstate the contribution of asthma to student absenteeism.

Accounting for student health and social factors, parents’ reports of their child ever having been diagnosed with asthma were not associated with absenteeism; this finding suggests that absenteeism among students with parent-reported asthma is partially explained by non–asthma-related factors such as transportation challenges, comorbid chronic mental and behavioral health conditions, or the stresses of poverty. Parents may be reporting very well controlled or quiescent asthma. In contrast, the relationships between student-reported asthma-related medical care and absenteeism and school health center record of asthma and absenteeism each persisted after adjusting for confounders. Student reports of using asthma-related health services (ED visits, hospitalization, medications) may be a better way to capture the true effect of asthma on student attendance than parent health forms.

Inconsistent with our hypothesis, accounting for various social factors did not attenuate the asthma–absenteeism relationship we observed. Homogeneity of the student population with respect to covariates could explain this pattern of results; however, we observed substantial variation in health and social conditions among students who were drawn from neighborhoods across the school district. It is also possible that unmeasured confounders such as parent agency and engagement explain part of this relationship.

Students with self-reported asthma medical care and a school health center record of asthma missed 1.9 and 1.5 extra days of school per year, respectively, compared with students without asthma. These estimates are comparable to those of studies that used school records to compare student attendance between students with and without asthma. For example, Silverstein et al examined attendance among 92 children with asthma compared with age- and sex-matched controls and found that students with asthma had 2.2 more days absent (21); Mizan et al found that 4th- and 5th-grade students (n = 914) had 1.7 excess absences (22); and Bonilla et al found that asthma was associated with 2 excess absences among children aged 5 to 7 years but not among those aged 8 to 11 years (12).

Our results suggest that improvements in asthma treatment and control could have a modest but meaningful impact on student absenteeism in this group at high demographic risk of poorer asthma control. Asthma based on student or health center records explained roughly 14% to 18% of all absenteeism among the 1,194 students in this urban school setting, which translates to 340 to 412 absences each year.

Many schools struggle to provide adequate asthma care with limited resources. Studies of school nursing care for asthma noted substantial gaps in available trained personnel and quality of care (23). Thus, opportunities to improve asthma care across settings remain. Moreover, it is important to note that, regardless of asthma, we found high levels of absenteeism overall (students were absent an average of 10.4 d/y). This finding is consistent with work that suggests that absenteeism is complex and multifactorial (24–27). Thus, while school-based asthma care has a substantial role to play, additional strategies to address the broader determinants of absenteeism are critical, particularly given the close links between absenteeism and student academic success (3).

The results of this study also inform efforts to better characterize the true population burden of childhood asthma, particularly in settings where asthma is expected to take the greatest toll on student health and achievement. Informed by literature suggesting that parent reports alone may underestimate asthma prevalence in schools that serve a substantial fraction of low-income students (11), we collected and compared asthma prevalence from several data sources reflecting a range of data that schools might have available (or could feasibly collect). Consistent with our hypothesis, estimated asthma prevalence was highest based on parent report (27%) and lowest based on student reports of asthma medication and ED use/hospitalization for asthma (15%). Parent-reported ever asthma prevalence was likely higher because it included students who had intermittent, quiescent, or well-controlled asthma, whereas student and health center records were more likely to identify those who required treatment.

Parent reports of asthma are the most common way that schools establish asthma prevalence among their students. However, more than 20% of students failed to return a school health form despite repeated mailings and forms sent home, parent reminders, and classroom competitions for form return. In settings where parent reports are incomplete and there is no school-based health center, a 2-question schoolwide asthma questionnaire may provide an inexpensive, feasible adjunct to parent reports to identify students with unmet care needs.

### Limitations

This study was conducted in 2 large schools with a high prevalence of asthma and absenteeism; therefore, the results may not be generalizable to other settings. However, they may provide insight into strategies where the need for intervention is most acute. Missing parent and student indicators of asthma were prevalent because of difficulty reaching parents and because of student and teacher absenteeism. We used rigorous analytic methods to account for potential biases related to missingness. Further, student-level data were not available for some covariates. Instead, we relied on census tract–level indicators, which could have introduced bias for students whose circumstances were substantially different from those of their neighbors. Unobserved confounders may have overestimated the role of asthma in student absenteeism. Moreover, reasons for student absences were not systematically collected. Therefore, we were unable to determine if a student’s absences were related directly to asthma or if they were student- or family-initiated (ie, absences) versus school-initiated (eg, suspensions). Finally, because this study focused on a population-based sample rather than a clinical sample of children with asthma, we did not have information about severity or persistence of asthma.

Despite these limitations, this study has several strengths. First, we had multiple indicators of asthma and detailed data on student comorbid health conditions and school district–reported attendance. In addition, by collecting data on the entire population at 2 urban schools with particularly high asthma burden, we were better able to estimate the extent to which school or public health system investments in improving asthma control would be expected to translate into better attendance in settings with similar asthma burden.

### Conclusions

It is essential to accurately characterize the role of student asthma in school absenteeism and to ensure that students with asthma are proactively identified and managed in partnership with schools. Parent report is the most common way that asthma prevalence is established by schools and local public health agencies; our results suggest that relying on parent reports alone may inflate the fraction of absenteeism that could be averted with better asthma identification and control. Schools with high asthma prevalence should therefore consider supplementing parent reports with student reports of asthma-related health care or SBHC records, if available.

The American Academy of Pediatrics Council on School Health has highlighted the important role of community-based medical homes in promoting school attendance among their pediatric patients by strengthening links between medical homes and school. Specifically, the Council recommends 1) asking about school and attendance, including about health problems contributing to absenteeism at every visit and communicating that information to school nurses; 2) establishing relationships with school nurses to improve chronic condition management; and 3) advocating for robust school health programs as part of the larger delivery system (28). Our results highlight the importance of funding and infrastructure to support high-quality school-based care, particularly for medically underserved students.

A lack of adequate school health staffing and capacity is a critical concern in many schools and districts nationwide. There are evidence-based strategies that may help reduce the burden of asthma, once identified, that require limited school health resources. Most notably, directly observed asthma controller therapy (DOT), which can be implemented by school health aides with proper training, may improve asthma control and facilitate greater partnership between community and school-based providers. Interventions such as school-based asthma DOT shift a common school health paradigm —often borne out of lean staffing — from reactivity to acute concerns to a more proactive preventive approach. This shift may make more efficient use of school health staff time because acute symptoms often require more time to address than administering controller medications. Multipronged case finding efforts in schools may result in improved access to, and adherence with, asthma care for vulnerable students, thereby improving health and reducing school absenteeism.

## Appendix

Sensitivity, Specificity, and Positive and Negative Prediction Rates for the 2 Student-Reported Asthma Screening Questions and Their Combination

**Table:**

Screening Question	Sensitivity	Specificity	Positive Prediction Rate	Negative Prediction Rate
Medication	0.671	0.829	0.510	0.905
Emergency department visit/hospitalization	0.630	0.840	0.511	0.895
Medication and emergency department visit/hospitalization	0.479	0.953	0.729	0.873
